# Is the lack of smartphone data skewing wealth indices in low-income settings?

**DOI:** 10.1186/s12963-021-00246-3

**Published:** 2021-02-01

**Authors:** Mathieu J. P. Poirier, Till Bärnighausen, Guy Harling, Ali Sié, Karen A. Grépin

**Affiliations:** 1 School of Global Health, Faculty of Health, York University, 4700 Keele Street, Dahdaleh Building 5022C, Toronto, Ontario M3J 1P3 Canada; 2 Global Strategy Lab, York University, 4700 Keele Street, Dahdaleh Building 5022C, Toronto, Ontario M3J 1P3 Canada; 3 Heidelberg Institute of Global Health (HIGH), Medical Faculty and University Hospital, Heidelberg University, Heidelberg, Germany; 4 Africa Health Research Institute (AHRI), Somkhele, KwaZulu-Natal South Africa; 5 MRC/Wits Rural Public Health and Health Transitions Research Unit (Agincourt), School of Public Health, Faculty of Health Sciences, University of the Witwatersrand, Johannesburg, South Africa; 6 Center for Population and Development Studies, Harvard University, Cambridge, MA USA; 7 Institute for Global Health, University College London, London, UK; 8 Department of Global Health and Population, Harvard T.H. Chan School of Public Health, Boston, USA; 9 Centre de Recherche en Santé de Nouna, Institut National de Santé Publique, Nouna, Burkina Faso; 10 School of Public Health, University of Hong Kong, Hong Kong, China

**Keywords:** Wealth index, Smartphones, Socioeconomic status, Principal components analysis, Burkina Faso, Health inequality, Development, Household expenditures, Education

## Abstract

**Background:**

Smartphones have rapidly become an important marker of wealth in low- and middle-income countries, but international household surveys do not regularly gather data on smartphone ownership and these data are rarely used to calculate wealth indices.

**Methods:**

We developed a cross-sectional survey module delivered to 3028 households in rural northwest Burkina Faso to measure the effects of this absence. Wealth indices were calculated using both principal components analysis (PCA) and polychoric PCA for a base model using only ownership of any cell phone, and a full model using data on smartphone ownership, the number of cell phones, and the purchase of mobile data. Four outcomes (household expenditure, education level, and prevalence of frailty and diabetes) were used to evaluate changes in the composition of wealth index quintiles using ordinary least squares and logistic regressions and Wald tests.

**Results:**

Households that own smartphones have higher monthly expenditures and own a greater quantity and quality of household assets. Expenditure and education levels are significantly higher at the fifth (richest) socioeconomic status (SES) quintile of full model wealth indices as compared to base models. Similarly, diabetes prevalence is significantly higher at the fifth SES quintile using PCA wealth index full models, but this is not observed for frailty prevalence, which is more prevalent among lower SES households. These effects are not present when using polychoric PCA, suggesting that this method provides additional robustness to missing asset data to measure underlying latent SES by proxy.

**Conclusions:**

The lack of smartphone data can skew PCA-based wealth index performance in a low-income context for the top of the socioeconomic spectrum. While some PCA variants may be robust to the omission of smartphone ownership, eliciting smartphone ownership data in household surveys is likely to substantially improve the validity and utility of wealth estimates.

## Introduction

The use of household assets to construct wealth indices has become a common method to measure socioeconomic status (SES) in low- and middle-income countries (LMICs) using household survey data. Researchers in the fields of health, economics, education, and public policy rely on wealth indices in settings where income and expenditure data may be unreliable or where household expenditure data are too difficult or resource-intensive to collect. Studies in a wide variety of settings have shown that the wealth index is consistently associated with income, expenditure, educational attainment, and health outcomes [1–4].

The household assets used to construct the wealth index have changed relatively little since the use of principal component analysis (PCA) was first popularized as a proxy for household wealth in the early 2000s. Indeed, the assets used by Filmer and Pritchett in their seminal article “Estimating Wealth Effects Without Expenditure Data—or Tears” are nearly the same as those used by researchers 20 years later [5]. Housing materials, drinking water source, sanitation facilities, cooking fuel, and ownership of household durables (such as bicycles, televisions, refrigerators, and cars) and agricultural assets (such as land and livestock ownership) remain the norm to this day [6–9].

If there is one asset class that has undergone a dramatic change in LMICs over the last two decades, it is undoubtedly cell phones [10]. Cell phones have transformed from a luxury item that only the most affluent households owned, to a ubiquitous and broadly affordable good for most households. As well, almost half of adults living in LMICs now report owning a smartphone, although disparities in access skew ownership to younger, wealthier, and more educated populations [11, 12]. With the advent of mobile data plans, cell phones have allowed millions to circumvent the need for a fixed telecommunication line to call, text, access the Internet, and have even replaced physical currency and in-person banking services in some countries [13–15].

Although most household surveys now include a binary question on whether households own a cell phone [16, 17], the lack of more detailed data on whether the cell phone is a smartphone, and whether the household has purchased mobile data may be depriving analysts of one of the most important modern social markers of wealth [11]. Notably, the only study we identified in a literature review of the implications of including smartphones in the construction of a wealth index advised against doing so because smartphone ownership data was only collected by 52 Demographic and Health Surveys (DHS), Multiple Indicator Cluster Surveys (MICS), and other national household surveys covering a population of 4,158,855 people, which fell short of their coverage benchmark of at least 75 countries and 3.5 billion people [16]. The question of whether this information should be more widely collected to meet this benchmark depends on whether the lack of smartphone data is skewing wealth indices in LMICs.

This study aims to evaluate the extent to which the lack of use of detailed data on smartphone ownership may affect the measurement of SES using wealth indices constructed from household survey data using a specially constructed survey module deployed in town of Nouna and surrounding villages, Burkina Faso. First, the paper will provide more context on Nouna and the survey. Second, it will then describe the methods used and present the main findings of the study, and finally, it will discuss this study’s limitations and conclude.

## Methods

This study was embedded within the Centre de Recherche en Santé de Nouna (CRSN)-Heidelberg Aging Study (CHAS), a cross-sectional household survey of older adults living in the Nouna department, which is located in northwest Burkina Faso near the border with Mali. CHAS was conducted in the CRSN Health and Demographic Surveillance Site (HDSS) [18], which provides ongoing, in-depth information about demographics and health of ~ 110,000 people in an area where vital registration systems are otherwise incomplete or absent.

The objective of the CHAS was to evaluate the risk factors for cardiovascular disease in a very low-income setting. The study sampled residents 40 years or older living in the Nouna HDSS, an estimated population of 18,000 adults at the time of the survey in 2018 [19, 20]. The survey targeted responses from 3000 adults using a multistage sampling strategy to select households: in the first stage, villages and towns were randomly selected, and then within each village, the study interviewed all adults when there were under 50 adults in a village, or up to 90 randomly selected adults when there were more than 50 adults in a village. The data collection period was between May and July 2018. Of the 3998 adults who were selected to participate in the study, 3033 could be located and consented to participate, and 3028 fully completed the expenditure module. After consent, all adults answered modules on their sociodemographic characteristics, physical health, cognition and mental health, health care, and the value of statistical life. In comparison to rural households in Burkina Faso, households in our sample are approximately the same size (8.1 vs. 7.9 members), and heads of household are older (54.8 vs. 47.0 years), more likely to be women (24.7% vs. 13.1%), and about as likely to have no formal education (84.4% vs. 88.0%) [21].

We used two different approaches to calculate wealth indices. The first followed the PCA approach described by Filmer and Pritchett and which is widely used to construct wealth indices and wealth quintiles using data collected in the DHS and MICS [5–7]. We also used polychoric PCA to construct asset indices because this method’s ability to make use of ordinal data and account for the lack of asset ownership may provide it superior robustness to missing smartphone data [22, 23]. Both indices were constructed using the same dataset, with ordinal asset data left in its original form for polychoric PCA analysis and dichotomized for PCA analysis.^1^ For both approaches, the analytical approach was to construct the wealth indices using a base model, which included only information on whether the household owned any cell phone or not, and a full model that included the same asset variables but also included data on the number of cell phones owned in a household, whether the household owned a smartphone, how many smartphones the household owned, and whether the household purchased mobile data. The four primary indices of interest were “base models” calculated using PCA and polychoric PCA with only binary cell phone ownership data, and “full models” calculated using both PCA and polychoric PCA but adding information on the number of cell phones and smartphones owned and the purchase of mobile data (Appendix Table 3).

Although wealth indices capture a longer-term measure of household SES than household income or expenditures [2], to validate the performance of the various indices, we used total household expenditures and education level as reference standards for all wealth indices. Expenditures were measured using a household consumption module and education was measured as an eight-level ordinal scale ranging from no formal education to college/university-level education. Two health outcomes were also used as outcome measures to explore differences in the strength of the association between wealth indices and better health outcomes. Based on previously published data from the CHAS [19, 20], frailty^2^ was selected as an outcome with higher prevalence among lower SES households, and diabetes^3^ was selected as an outcome with higher prevalence among higher SES households.

Descriptive tables were used to explore asset ownership among households with a cell phone and with a smartphone, and kernel-weighted local polynomial plots were used to compare household expenditures at each wealth quintile for base indices and full indices. Since raw wealth index scores lack a meaningful scale, we used rank-based nonparametric Spearman correlation coefficients to evaluate the strength of association between wealth indices and household expenditures [24].

Next, a series of regression-based methods were used to evaluate the significance of the effect of including more detailed cell phone data. Separate ordinary least squares (OLS) regressions of base model wealth index quintiles and full model wealth index quintiles were each run against two outcome variables of the log of monthly household expenditure and education level using the poorest wealth index quintile as the omitted comparison group. Sensitivity tests were also conducted to examine the independent effects of adding each new household asset variable to the calculation of PCA-derived and polychoric PCA-derived wealth indices, and of normalizing the number of cell phones and smartphones owned by the number of adults in the household.^4^

Logistic regressions of base model wealth index quintiles and full model wealth index quintiles were also run against two dichotomous outcome variables of diabetes prevalence and frailty prevalence. The poorest wealth index quintile was used as the omitted comparison group for diabetes prevalence and the richest wealth index quintile was used for frailty prevalence to capture changes population health in the quintiles of interest for each outcome. Wald tests were then run to compare coefficients derived from the base and full models, and all analyses were repeated for both PCA-derived and polychoric PCA-derived wealth indices^5^ [25]. A rejection of the null hypothesis that base model wealth indices and full model wealth indices differ at any given quintile would require a Wald test *p* value of less than 0.05, meaning that a quintile-specific difference between the base and full models was identified.

As a final analysis, an OLS regression was used to evaluate whether household expenditures were significantly associated with a shift of wealth quintile from base to full model for both PCA and polychoric PCA. If the rearrangement of wealth index quintiles were purely random, we would not expect a change in household expenditure to be significantly associated with a change in quintile. Together, these methods quantify whether asset ownership differs among smartphone owners; how associations between wealth indices and expenditure, education, and health are mediated by changes induced by including smartphone data; and whether rearrangement is non-random.

## Results

**Fig. 1:**
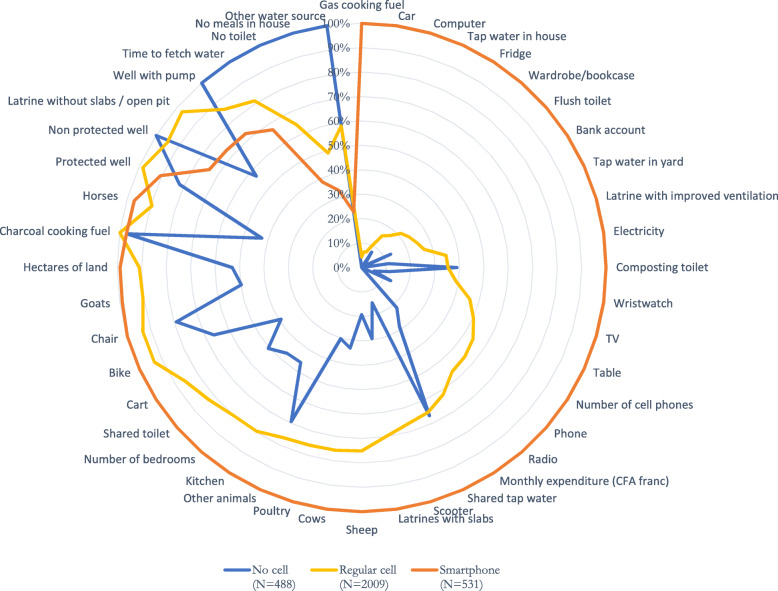
Radar chart of the prevalence of normalized asset ownership for households with no cell phone, a regular cell phone, or a smartphone. All figures are scaled to the highest value for that asset and more detailed information is available in Appendix Table 3

Households that own a smartphone (17.5%) reported noticeably different asset ownership than those that own a regular cell phone (66.4%) or those that do not own a cell phone (16.1%). Descriptive data for every asset variable included in wealth index calculation is detailed in Fig. 1,^6^ where a pattern approximating a dose-response or social gradient from no cell phone to regular cell phone to smartphone ownership emerges. For example, households without a cell phone report monthly expenditure of 16,748 CFA francs (€25.66), while the equivalent figure for households with a regular cell phone is 44,691 CFA francs (€68.48), and 58,726 CFA francs (€89.99) for households with a smartphone (Appendix Table 3). Conversely, 73.0% of households without a cell phone do not have a toilet, while that figure drops to 46.9% for households with a regular cell phone, and 28.1% for households with a smartphone. Although not conclusive, these differences support the plausibility of the hypothesis that missing smartphone data may skew wealth index construction.

**Fig. 2:**
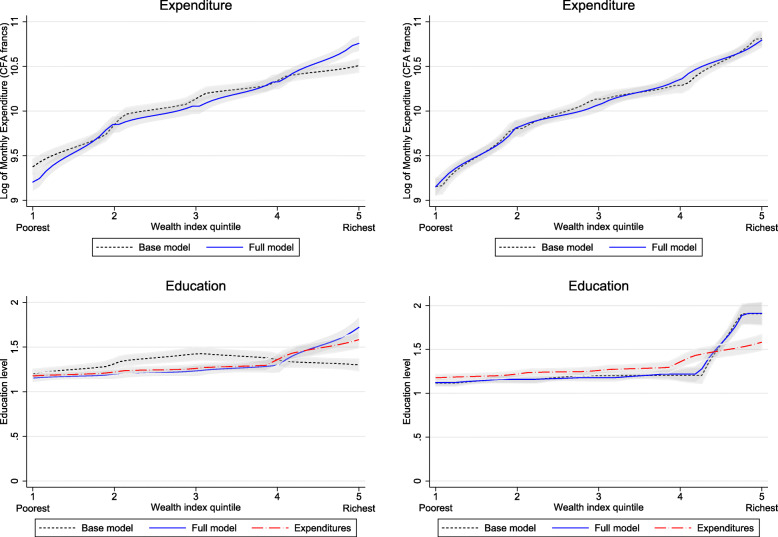
Kernel-weighted local polynomial plots of log of monthly household expenditures (in CFA francs) for each wealth quintile (top) and education level (bottom) for base and full model using PCA (left) and polychoric PCA (right). Base model refers to wealth index calculated using only information on whether the household owned any cell phone or not, and a full model refers to wealth index calculated with the addition of data on the number of cell phones owned in a household, whether the household owned a smartphone, how many smartphones the household owned, and whether the household purchased mobile data

**Fig. 3:**
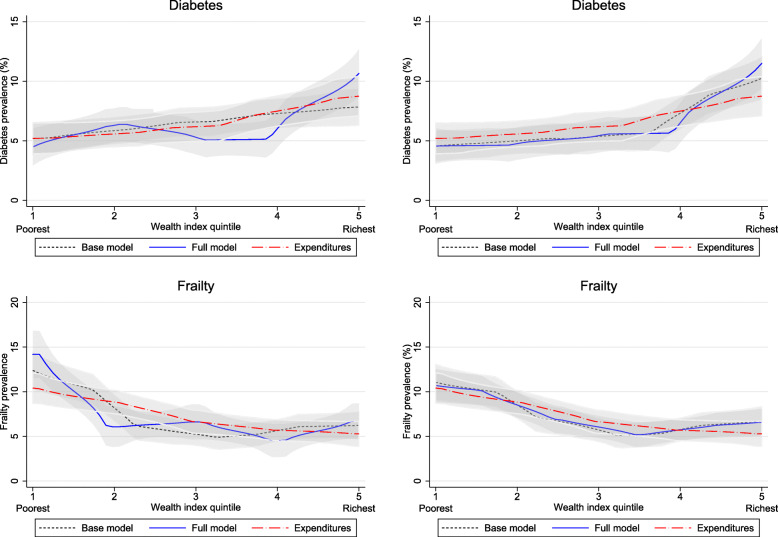
Kernel-weighted local polynomial plots of prevalence of diabetes (top) and frailty (bottom) by base model quintile, full model quintile, and household expenditure quintile using PCA (left) and polychoric PCA (right). Base model refers to wealth index calculated using only information on whether the household owned any cell phone or not, and a full model refers to wealth index calculated with the addition of data on the number of cell phones owned in a household, whether the household owned a smartphone, how many smartphones the household owned, and whether the household purchased mobile data

Comparing plots of base model wealth indices (i.e., no smartphone data) with full models (i.e., all smartphone data) begins to reveal a divergence between the performance of PCA-derived wealth indices and polychoric PCA-derived wealth indices. This divergence can be observed in Fig. 2, with both household expenditure and education level appearing to be significantly higher at the fifth (richest) SES quintile of the full model wealth index constructed using PCA, but with no significant difference observed for the wealth index constructed using polychoric PCA. Similar patterns can be observed in Fig. 3, with suggestive differences between the fifth quintile of base and full model wealth indices for diabetes prevalence, but no similar divergence evident for the polychoric PCA indices. Interestingly, this divergence is not readily apparent for the outcome of frailty, which is more prevalent among the first (poorest) SES quintile.

These differences are confirmed by regression analysis and Wald tests reported in Table 1. Expenditures are significantly and increasingly larger than the lowest SES quintile for every higher quintile using both the base and full model; however, the full index coefficient of 1.55 for the fifth quintile is significantly higher than the 1.26 obtained in the base model for the PCA-derived wealth index. The polychoric PCA-derived wealth indices are also significantly and increasingly larger with every increase in SES quintile, but the fifth quintile coefficients of 1.66 and 1.64 for the base and full models are nearly identical. This is also supported by comparing the divergence in Spearman correlation coefficients for household expenditure with the base model (0.38) and full model (0.47) for PCA wealth indices, while no significant difference can be observed for household expenditure and polychoric PCA base model (0.49) and full model (0.49) (Appendix Tables 4 and 5).

**Table 1:** OLS regression of base and full model wealth index quintiles with log of household expenditures and education level (beta coefficients reported), logistic regression of base and full model wealth index quintiles with diabetes and frailty prevalence (odds ratios reported), lower (LCI) and upper (UCI) 95% confidence intervals, chi^2^, and *p* values for Wald tests comparing coefficients derived from base and full models. All analyses are reported for both PCA-derived and polychoric PCA-derived wealth indices

Outcome	Quintile	Base	LCI	UCI	Full	LCI	UCI	chi^**2**^	Prob > chi^**2**^
**PCA wealth index**									
**Expenditures**	1	-	-	-	-	-	-	-	-
	2	0.61**	0.47	0.75	0.64**	0.51	0.78	0.43	0.51
	3	0.87**	0.74	1.00	0.85**	0.72	0.98	0.16	0.69
	4	1.06**	0.93	1.19	1.12**	1.00	1.25	1.70	0.19
	5	1.26**	1.13	1.40	1.55**	1.42	1.68	43.57**	0.00
**Education**	1	-	-	-	-	-	-	-	-
	2	0.13**	0.04	0.21	0.04	-0.02	0.10	4.98*	0.03
	3	0.26**	0.15	0.37	0.08*	0.01	0.15	11.38**	0.00
	4	0.17**	0.08	0.26	0.14**	0.06	0.23	0.26	0.61
	5	0.11*	0.02	0.19	0.57**	0.44	0.70	68.76**	0.00
**Diabetes**	1	-	-	-	-	-	-	-	-
	2	1.37	0.81	2.31	1.78*	1.05	3.01	1.91	0.17
	3	1.53	0.92	2.54	1.26	0.72	2.20	1.00	0.32
	4	1.52	0.91	2.54	1.30	0.75	2.27	0.56	0.45
	5	1.97**	1.21	3.21	3.15**	1.93	5.13	10.54**	0.00
**Frailty**	1	2.44**	1.61	3.69	2.30**	1.53	3.47	0.23	0.63
	2	1.21	0.76	1.92	0.90	0.56	1.46	2.90	0.09
	3	0.65	0.38	1.10	0.99	0.62	1.58	2.92	0.09
	4	0.93	0.57	1.51	0.66	0.39	1.11	1.49	0.22
	5	-	-	-	-	-	-	-	-
**Polychoric PCA wealth index**									
**Expenditure**	1	-	-	-	-	-	-	-	-
	2	0.65**	0.52	0.78	0.66**	0.53	0.80	0.09	0.77
	3	0.98**	0.85	1.11	0.92**	0.79	1.05	2.67	0.10
	4	1.13**	1.00	1.27	1.21**	1.08	1.33	3.47	0.06
	5	1.66**	1.53	1.79	1.64**	1.51	1.78	0.21	0.65
**Education**	1	-	-	-	-	-	-	-	-
	2	0.04	− 0.06	0.15	0.04	− 0.07	0.14	0.14	0.71
	3	0.08	− 0.02	0.19	0.06	− 0.05	0.16	2.20	0.14
	4	0.09	− 0.02	0.19	0.10	− 0.01	0.20	0.19	0.67
	5	0.79**	0.69	0.89	0.79	0.69**	0.89	0.00	0.99
**Diabetes**	1	-	-	-	-	-	-	-	-
	2	1.30	0.75	2.25	1.03	0.59	1.79	0.88	0.35
	3	1.20	0.69	2.09	1.23	0.72	2.09	0.02	0.89
	4	1.52	0.90	2.59	1.27	0.75	2.15	1.15	0.28
	5	3.16**	1.96	5.11	3.01**	1.89	4.79	0.14	0.71
**Frailty**	1	1.77**	1.17	2.68	1.70*	1.12	2.57	0.41	0.52
	2	1.25	0.81	1.94	1.21	0.78	1.87	0.15	0.70
	3	0.65	0.39	1.08	0.68	0.42	1.12	0.09	0.76
	4	0.79	0.49	1.28	0.71	0.44	1.16	0.71	0.40
	5	-	-	-	-	-	-	-	-

* denotes statistical significance at the p < 0.05 level

** denotes statistical significance at the p <0.01 level

This pattern is repeated and even more pronounced for the outcome of education level, which is significantly higher than the lowest quintile at every higher level for the base PCA index, but only for the top three quintiles in the full model. This results in the base model having a significantly higher education level than the full model at the third (middle) quintile, and the full model having a significantly higher education level at the fifth (richest) quintile. In contrast, the polychoric PCA base and full models are nearly identical, and only significantly different than the first quintile at the fifth (richest) quintile level.

This pattern of divergence is nearly identical to that obtained for the outcome of diabetes, which is more prevalent among higher SES households. Odds ratios for diabetes using PCA-derived wealth indices are not statistically different for the first four quintiles, but at the fifth quintile odds ratios become significantly larger for the full model (3.15) than the base model (1.97). Like household expenditures, odds ratios for the polychoric PCA base model and full models are not statistically different at any quintile level. Conversely, odds ratios for frailty are not significantly different between the full model and base model at any SES quintile level for either PCA- or polychoric PCA-derived wealth indices.

Sensitivity tests reported in Appendix Table 6 demonstrate that the independent addition of smartphone ownership, purchase of mobile data, number of cell phones, or number of smartphones cause significant changes at the upper end of the PCA wealth index, and that inclusion of all variables results in the largest changes to both expenditure and education distribution. Finally, normalizing the number of cell phones and smartphones owned per household member negates the significant effect found for adding only the number of cell phones owned, but does not impact the model adding the number of smartphones owned or the full model (Appendix Table 7).

Finally, analysis of households that shift at least one quintile after incorporating smartphone data reveals greater reorganization at the upper end of the socioeconomic spectrum for PCA wealth indices (Appendix Table 8) and more stability for the polychoric PCA wealth indices (Appendix Table 9). An OLS regression of the change in monthly household expenditure for every shift in wealth index quintile reveals an increase of 17,473 CFA francs (€26.77) for households moving up one quintile and an increase of 55,923 CFA francs (€85.69) for households shifting up two quintiles from base to full models for PCA wealth indices (Table 2). For polychoric PCA wealth indices, not only do no households shift down more than one quintile or up more than two quintiles, but there is no significant difference in household expenditure at any level of quintile shift.

**Table 2:** OLS regression of change in monthly household expenditures in CFA francs by shift in wealth index quintile for both PCA and polychoric PCA wealth indices

	PCA wealth index	Polychoric PCA wealth index
**Decrease of 2**	8,634	N/A
	(− 101,449 to 118,718)	
**Decrease of 1**	− 634.9	− 6694
	(− 6133 to 4863)	(− 14,000 to 611.2)
**No change**	omitted	omitted
**Increase of 1**	17,473**	− 4616
	(10,931 to 24,015)	(− 12,027 to 2795)
**Increase of 2**	55,923**	11,939
	(43,072 to 68,773)	(− 43,965 to 67,843)
**Increase of 3**	22,793	N/A
	(− 22,202 to 67,788)	

* denotes statistical significance at the p < 0.05 level

** denotes statistical significance at the p <0.01 level

## Discussion

In a cross-sectional sample of households in Nouna, Burkina Faso, we find that the inclusion of additional information on cell phone ownership leads to significant changes in the estimates of the wealth index. There is a significant shift concentrated at the top of the socioeconomic spectrum using the traditional PCA approach, but not for the polychoric PCA approach, which is robust to the lack of smartphone data. These results would imply that wealth indices may be skewed for the hundreds of current and planned household surveys that make use of a PCA approach to construct wealth indices, in particular as smartphone ownership becomes more common throughout LMICs [11].

The evidence produced in this study aligns with the expected impact of smartphone ownership as published in academic journals and technical reports. Although some authors have hypothesized that the lack of smartphone data may be affecting the performance of wealth indices [26, 27], to our knowledge, none have empirically tested the extent of the effect in the field. Nevertheless, as surveys begin to collect smartphone ownership data for use in wealth index calculation, it is becoming clear that ownership is becoming more common throughout every region of the world and that ownership is more common among relatively wealthy households [11, 28, 29].

Although we are unable to generalize findings to other contexts, our in-depth focus of one study site does provide this study with many strengths. The survey instrument was specifically designed to address this research question by collecting data on household assets, two modules measuring monthly expenditures, and validated instruments measuring health outcomes including frailty and diabetes. We were also able to demonstrate that the shift in households at the top end of the wealth index was not limited to associations with household expenditure but were also linked to education and health indicators. Wealth-based inequalities in frailty (which is concentrated among relatively poor households) were unaffected by the changes to wealth index after including data on smartphone ownership, while inequalities in diabetes (which is concentrated among relatively wealthy households) were significantly affected in PCA-derived wealth indices. Finally, the more pronounced social gradient in education attainment found in the full PCA wealth index and both polychoric PCA wealth indices provide evidence that wealth indices calculated with standard PCA that lack smartphone data—as is the current standard—are the least plausible proxies for SES among the four options evaluated in this study.

Our findings indicating that the polychoric PCA method of constructing wealth indices proved more robust to missing smartphone data than regular PCA suggests that the choice of method for constructing wealth indices may be quite consequential in the context of missing data for socially valued household goods. The debate over whether the PCA method of constructing wealth indices should be revisited has centered on its inability to quantify directionality in ordinal data, its insensitivity to the lack of household goods, and potential lack of robustness to missing variables [22, 30, 31]. Despite these concerns, many studies have found that, in practice, there is often little difference in the performance of alternate methods for wealth index construction, including polychoric PCA [1–4]. Our findings, however, suggest that the additional robustness provided by using polychoric PCA may become increasingly important in the context of missing data on goods that underlie the latent SES spectrum that wealth indices are measuring by proxy.

The primary limitations of this study lie in our inability to generalize findings to other contexts. The World Bank identifies Burkina Faso as the 14th poorest country in the world in terms of GDP per capita, and as a town of approximately 30,000 people, Nouna does not have the same degree of access to technology as more populated and wealthier cities [32]. We can presume that in many contexts, smartphone ownership is already more common than we observed at this study site. If so, smartphones may be less of a marker of high social status, and as an increasingly affordable and necessary good for the middle-class, the lack of smartphone ownership may soon represent a marker of lower social status. If so, our findings that lack of smartphone ownership primarily affects the upper end of the socioeconomic spectrum may be reversed in contexts where smartphone ownership is common. Finally, heads of household in our sample are older and more likely to be female than the general rural population of Burkina Faso. Since smartphone ownership is more common among younger populations and men [11, 33, 34], this may indicate that our findings underestimate the magnitude of smartphones’ impact on wealth index measurement.

In conclusion, this study provides the first empirical evidence quantifying the impact of the lack of smartphone data on the measurement of SES with household surveys in low- and middle-income countries. Missing smartphone data skewed the wealthiest quintile of PCA-derived wealth indices when compared to household expenditures, education level, and health outcomes, but this divergence was not present using polychoric PCA-derived wealth indices. While more study is needed to evaluate the generalizability of these findings, this study suggests that international household surveys should strongly consider the regular and standardized collection of data on smartphone ownership, purchase of cellular data, and number of cell phones and smartphones owned. At minimum, the addition of binary variables on smartphone ownership and mobile data purchase appear to reliably improve the performance of the standard wealth index across outcomes and sensitivity tests. Not only would this easily implemented and inexpensive adaptation support more reliable calculation of wealth indices, it would offer added benefits of informing intervention planning using this emerging channel to reach households with messaging, training activities, social media engagement, and smartphone applications to promote health in contexts around the world.

## Data Availability

Data are not publicly available as consent was not given by participants for data to be shared openly. This is in part because entire age cohorts of some villages are included in the dataset, potentially allowing for deductive disclosure with sufficient local information. For this reason, anonymized data is available from CHAS study data controllers only following signature of a data use agreement restricting onward transmission. Anyone wishing to replicate the analyses presented, or conduct further collaborative analyses using CHAS (which are welcomed and considered based on a letter of intent), should contact Dr Guy Harling (g.harling@ucl.ac.uk) in the first instance.

## Appendix

**Table 3:** Sample sizes and distributions by ownership of no cell phone, all cell phones, regular cell phones, smartphones, and all households for all wealth index variables

Asset class	Asset	N	No cell (***N*** = 488)	All cells (***N*** = 2540)	Regular cell (***N*** = 2009)	Smartphone (***N*** = 531)	Overall (***N*** = 3028)
Agriculture	Hectares of land	2989	2.28	4.30	3.91	4.25	3.97
Agriculture	Cows	3026	1.49	3.97	3.38	4.47	3.57
Agriculture	Horses	3026	0.51	1.19	1.06	1.15	1.08
Agriculture	Goats	3027	2.03	4.08	3.68	4.03	3.75
Agriculture	Sheep	3021	0.72	3.42	2.82	3.76	2.98
Agriculture	Other animals	3028	0.37	0.44	0.41	0.53	0.43
Agriculture	Poultry	3026	3.49	10.31	8.73	11.50	9.21
Community	Electricity	3028	3.5%	16.6%	10.9%	31.3%	14.5%
Community	Bank account	3028	0.4%	12.2%	6.5%	28.1%	10.3%
Cooking fuel	Gas	3028	0.0%	0.9%	0.2%	3.8%	0.8%
Cooking fuel	Wood charcoal	3028	94.3%	97.0%	97.0%	94.4%	96.6%
Cooking fuel	No meals in house	3028	5.7%	2.0%	2.8%	1.9%	2.6%
Durables	Number of cell phones	3026	0.00	2.80	2.05	3.78	2.35
Durables	Owns smartphone	3028	0.0%	20.9%	0.0%	100.0%	17.5%
Durables	Smartphones owned	3028	0.00	0.39	0.00	1.87	0.33
Durables	Buys mobile data	3028	0.0%	5.4%	0.0%	25.6%	4.5%
Durables	Radio	3028	12.9%	42.6%	33.3%	58.9%	37.8%
Durables	TV	3028	2.5%	30.5%	21.5%	46.7%	26.0%
Durables	Phone	3028	0.0%	1.1%	0.8%	1.5%	1.0%
Durables	Computer	3028	0.0%	2.2%	0.5%	7.9%	1.8%
Durables	Fridge	3028	0.8%	3.8%	1.7%	10.9%	3.3%
Durables	Table	3028	4.3%	22.4%	16.6%	33.0%	19.5%
Durables	Chair	3028	76.2%	94.0%	90.0%	96.4%	91.1%
Durables	Wardrobe/bookcase	3028	0.4%	6.1%	2.8%	16.2%	5.2%
Durables	Watch	3028	3.1%	15.1%	10.3%	26.6%	13.2%
Durables	Bike	3028	62.5%	93.8%	87.7%	94.0%	88.8%
Durables	Scooter	3028	10.9%	60.2%	48.1%	71.9%	52.2%
Durables	Cart	3028	24.8%	62.0%	54.4%	63.3%	56.0%
Durables	Car	3028	0.0%	1.2%	0.3%	4.3%	1.0%
Housing	Kitchen	3028	36.9%	72.1%	63.6%	79.8%	66.4%
Housing	Number of bedrooms	3025	1.84	3.58	3.16	3.95	3.30
Sanitation	Shared toilet	3028	16.2%	29.6%	26.5%	32.0%	27.4%
Sanitation	Flush toilet	3028	0.0%	0.5%	0.2%	1.1%	0.4%
Sanitation	Latrine with improved ventilation	3028	0.0%	0.4%	0.2%	0.8%	0.3%
Sanitation	Latrines with slabs	3028	14.3%	41.0%	34.1%	48.8%	36.7%
Sanitation	Latrine without slabs / open pit	3028	8.8%	15.4%	15.0%	11.3%	14.4%
Sanitation	Composting toilets	3028	3.9%	4.8%	3.5%	10.0%	4.7%
Sanitation	No toilet	3028	73.0%	38.0%	46.9%	28.1%	43.6%
Water source	Time to fetch water	3024	15.8	11.8	12.8	10.6	12.4
Water source	Tap water in house	3028	0.0%	2.0%	0.6%	6.8%	1.7%
Water source	Tap water in yard	3028	0.6%	2.0%	1.2%	4.7%	1.8%
Water source	Shared tap water	3028	9.4%	10.2%	9.2%	14.1%	10.1%
Water source	Well with pump	3028	23.4%	18.7%	20.0%	16.9%	19.5%
Water source	Protected well	3028	24.4%	29.8%	29.3%	26.9%	28.9%
Water source	Non protected well	3028	39.3%	35.1%	37.2%	29.2%	35.8%
Water source	Protected source	3028	1.0%	0.4%	0.6%	0.2%	0.5%
Water source	Non protected source	3028	0.2%	1.0%	0.9%	0.8%	0.9%
Water source	Other water source	3028	1.6%	0.7%	1.0%	0.4%	0.9%
Expenditure	Monthly expenditure (CFA franc)	3028	16748	44691	36246	58726	40188

**Table 4:** Spearman correlation coefficients for each wealth index variation calculated using PCA

	HH expenditure	Base model	+ Number of cells	+ Smartphone dummy	+ Number of smartphones	+ Smartphone and data dummies	Full model
**HH expenditure**	1						
**Base model**	0.377	1					
**+ Number of cells**	0.4159	0.9901	1				
**+ smartphone dummy**	0.3981	0.9963	0.9955	1			
**+ Number of smartphones**	0.451	0.9554	0.9835	0.9753	1		
**+Smartphone & data dummies**	0.4215	0.9829	0.9936	0.9945	0.9893	1	
**Full model**	0.4651	0.9299	0.9661	0.9551	0.9958	0.9765	1

**Table 5:** Spearman correlation coefficients for each wealth index variation calculated using polychoric PCA (a model using smartphone and data dummy variables only could not reach convergence using polychoric PCA)

	HH expenditure	Base model	+ Number of cells	+ smartphone dummy	+ Number of smartphones	Full model
**HH expenditure**	**1**					
**Base model**	0.4886	1				
**+ Number of cells**	0.4931	0.9917	1			
**+ smartphone dummy**	0.4897	0.9937	0.9863	1		
**+ Number of smartphones**	0.4945	0.9883	0.9976	0.9898	1	
**Full model**	0.4944	0.9851	0.9952	0.9887	0.9985	1

**Table 6:** OLS regression of base and alternative model wealth index quintiles with log of household expenditures and education level (beta coefficients reported, chi^2^, and *p* values for Wald tests comparing coefficients derived from base and comparison models). Base model contains only binary cell phone data, alternative wealth index (alt WI) model 1 adds binary smartphone ownership, alt WI model 2 adds binary mobile data, alt WI 3 adds number of cell phones, alt WI 4 adds number of cell phones and number of smartphones (for polychoric PCA alternate model 4 only, binary cell phone was dropped from analysis to allow model to converge), alt WI 5 adds binary smartphone ownership and mobile data, and the full model has data on the number of cell phones, number of smartphones, and mobile data. All analyses are reported for both PCA-derived and polychoric PCA-derived wealth indices

	PCA										Polychoric PCA									
	Expenditure					Education					Expenditure					Education				
Quintile	1	2	3	4	5	1	2	3	4	5	1	2	3	4	5	1	2	3	4	5
Base WI	-	0.61	0.87	1.06	1.26	-	0.13	0.26	0.17	0.11	-	0.65	0.98	1.13	1.66	-	0.04	0.08	0.09	0.79
Alt WI 1	-	0.63	0.85	1.07	1.34	-	0.11	0.21	0.21	0.20	-	0.69	0.93	1.18	1.66	-	0.05	0.06	0.11	0.80
Chi^2^	-	0.77	0.43	0.34	18.27**	-	0.95	2.56	1.03	15.09**	-	1.90	4.00	2.92	0.02	-	0.70	4.37*	1.56	0.30
*p* value	-	0.38	0.51	0.56	0.00	-	0.33	0.11	0.31	0.00	-	0.17	0.05	0.09	0.88	-	0.40	0.04	0.21	0.58
Alt WI 2	-	0.62	0.86	1.09	1.31	-	0.09	0.19	0.24	0.17	-	0.57	0.90	1.10	1.59	-	0.04	0.06	0.10	0.80
Chi^2^	-	0.34	0.19	2.74	14.15**	-	3.41	3.86	3.71	10.82**	-	2.45	4.48*	0.97	4.01	-	0.09	3.41	0.37	0.22
*p* value	-	0.56	0.66	0.10	0.00	-	0.07	0.05	0.05	0.00	-	0.12	0.03	0.33	0.05	-	0.76	0.06	0.54	0.64
Alt WI 3	-	0.66	0.85	1.12	1.40	-	0.06	0.21	0.24	0.20	-	0.64	0.93	1.21	1.62	-	0.04	0.05	0.09	0.77
Chi^2^	-	2.18	0.51	3.22	32.79**	-	6.73*	1.83	2.48	11.65**	-	0.02	2.27	4.60*	1.62	-	0.15	6.12*	0.20	1.44
*p* value	-	0.14	0.47	0.07	0.00	-	0.01	0.18	0.12	0.00	-	0.88	0.13	0.03	0.20	-	0.70	0.01	0.66	0.23
Alt WI 4	-	0.68	0.86	1.15	1.53	-	0.03	0.09	0.18	0.44	-	0.67	0.94	1.23	1.64	-	0.03	0.06	0.08	0.77
Chi^2^	-	2.31	0.07	4.46*	47.70**	-	8.46**	10.61**	0.09	44.80**	-	0.13	1.25	5.75*	0.19	-	0.62	1.88	0.01	0.84
*p* value	-	0.13	0.79	0.03	0.00	-	0.00	0.00	0.77	0.00	-	0.72	0.26	0.02	0.66	-	0.43	0.17	0.93	0.36
Alt WI 5	-	0.67	0.84	1.10	1.46	-	0.06	0.16	0.16	0.37	-	0.63	0.89	1.12	1.63	-	0.04	0.04	0.10	0.80
Chi^2^	-	3.02	1.05	1.15	43.10**	-	4.83*	4.93*	0.05	34.71**	-	0.12	4.58*	0.07	0.63	-	0.08	6.28*	0.37	0.50
*p* value	-	0.10	0.30	0.28	0.00	-	0.03	0.03	0.82	0.00	-	0.73	0.03	0.80	0.43	-	0.78	0.01	0.54	0.48
Full WI	-	0.64	0.85	1.12	1.55	-	0.04	0.08	0.14	0.57	-	0.66	0.92	1.21	1.64	-	0.04	0.06	0.10	0.79
Chi^2^	-	0.43	0.16	1.70	43.57**	-	4.98*	11.38**	0.26	68.76**	-	0.09	2.67	3.47	0.21	-	0.14	2.20	0.19	0.00
*p* value	-	0.51	0.69	0.19	0.00	-	0.03	0.00	0.61	0.00	-	0.77	0.10	0.06	0.65	-	0.71	0.14	0.67	0.99

* denotes statistical significance at the p < 0.05 level

** denotes statistical significance at the p <0.01 level

**Table 7:** OLS regression of base and alternative model wealth index quintiles normalizing the number of cell phones and smartphones owned to the number of adults in the household with log of household expenditures and education level (beta coefficients reported, chi^2^, and *p* values for Wald tests comparing coefficients derived from base and comparison models). Base model contains only binary cell phone data, alternative wealth index (alt WI) model 1 adds number of cell phones, alt WI 2 model adds number of cell phones and number of smartphones (for polychoric PCA alt WI 2 model only, binary cell phone was dropped from analysis to allow model to converge), and the Alt full model has data on the number of cell phones, number of smartphones, and mobile data. All analyses are reported for both PCA-derived and polychoric PCA-derived wealth indices

	PCA										Polychoric PCA									
	Expenditure					Education					Expenditure					Education				
Quintile	1	2	3	4	5	1	2	3	4	5	1	2	3	4	5	1	2	3	4	5
Base WI	-	0.61	0.87	1.06	1.26	-	0.13	0.26	0.17	0.11	-	0.65	0.98	1.13	1.66	-	0.04	0.08	0.09	0.79
Alt WI 1	-	0.67	0.86	1.07	1.29	-	0.14	0.28	0.15	0.11	-	0.64	0.94	1.15	1.61	-	0.04	0.05	0.10	0.79
Chi^2^	-	4.27*	0.03	0.21	3.30	-	0.40	1.22	0.86	0.00	-	0.03	0.68	0.26	2.64	-	0.00	3.82	0.41	0.06
Prob > chi^2^	-	0.04	0.85	0.64	0.07	-	0.53	0.27	0.35	0.98	-	0.87	0.41	0.61	0.10	-	0.95	0.05	0.52	0.80
Alt WI 2	-	0.68	0.88	1.10	1.47	-	0.07	0.15	0.17	0.41	-	0.64	0.97	1.14	1.63	-	0.03	0.04	0.12	0.78
Chi^2^	-	2.93	0.11	0.93	38.29**	-	3.04	5.04*	0.01	41.58**	-	0.06	0.04	0.06	1.10	-	0.36	5.76*	1.97	0.14
Prob > chi^2^	-	0.09	0.74	0.33	0.00	-	0.08	0.02	0.92	0.00	-	0.80	0.84	0.80	0.29	-	0.55	0.02	0.16	0.70
Alt Full	-	0.64	0.87	1.12	1.51	-	0.04	0.10	0.13	0.53	-	0.65	0.94	1.15	1.62	-	0.02	0.05	0.09	0.81
Chi2	-	0.40	0.00	1.70	33.22**	-	5.02	9.26*	0.51	61.85**	-	0.00	1.14	0.15	1.36	-	1.06	4.61*	0.00	0.74
Prob > chi^2^	-	0.53	1.00	0.19	0.00	-	0.03	0.00	0.47	0.00	-	0.99	0.28	0.70	0.24	-	0.30	0.03	0.98	0.39

* denotes statistical significance at the p < 0.05 level

** denotes statistical significance at the p <0.01 level

**Table 8:** Quintile shifts from base PCA wealth index to full PCA wealth index

		Full model				
		1	2	3	4	5
**Base model**	**1**	536	62	4	0	0
	**2**	66	428	82	20	6
	**3**	0	112	365	73	52
	**4**	0	0	150	342	110
	**5**	0	0	1	167	434

**Table 9:** Quintile shifts from base polychoric PCA wealth index to full polychoric PCA wealth index

		Full model				
		1	2	3	4	5
**Base model**	**1**	544	58	0	0	0
	**2**	58	480	63	1	0
	**3**	0	64	462	73	3
	**4**	0	0	77	469	56
	**5**	0	0	0	59	543

## Abbreviations

CHAS: Centre de Recherche en Santé de Nouna Heidelberg Aging Study
CRSN: Centre de Recherche en Santé de Nouna
DHS: Demographic and Health Surveys
HDSS: Health and Demographic Surveillance Site
LMIC: Low- and middle-income country
MICS: Multiple Indicator Cluster Surveys
OLS: Ordinary least squares
PCA: Principal component analysis
SES: Socioeconomic status

## Footnotes

1 The native Stata pca (principal component analysis) command with one component (eigenvector) retained was used to calculate PCA wealth indices, and the polychoricpca user-written Stata command with one score (eigenvector) retained was used to calculate polychoric PCA wealth indices.

2 As measured by the Fried frailty index [11]

3 As defined as either self-reported diagnosis; being on treatment; a non-fasting point of care capillary glucose level > 200 mg/dL, HbA1c> 6.5% or fasting glucose > 126 mg/dL

4 Our primary models do not normalize the number of cell phones or smartphones owned by the number of adults in the household. This is because knowledge users would likely choose to analyze these assets in the same way as agricultural assets because multiple cell phones are often owned by one household member.

5 As calculated through Stata seemingly unrelated estimation (suest) postestimation command

6 Table 1 presents data normalized to the maximum value of each asset for ease of presentation. Full raw data is presented in Appendix Table 3.
